# Photoluminescence Properties of Lignin With a Genetically Introduced Luminophore in a Transgenic Hybrid Aspen That Overproduces Feruloyl‐CoA 6′‐Hydroxylase

**DOI:** 10.1111/pbi.70390

**Published:** 2025-10-03

**Authors:** Masatsugu Takada, Shota Horinouchi, Naning Wang, Mikiko Uesugi, Shinya Kajita

**Affiliations:** ^1^ Graduate School of Bio‐Applications and Systems Engineering (BASE), Tokyo University of Agriculture and Technology Koganei Tokyo Japan; ^2^ Graduate School of Agriculture Ehime University Matsuyama Ehime Japan

**Keywords:** coumarins, genetic modification, hybrid aspen, lignin modification, molar mass distribution, photo‐dimerisation, photoluminescence, scopoletin

## Abstract

Lignin, a major cell‐wall component of woody biomass, exhibits photoluminescent (PL) properties. Controlling the intensity and colour of the PL is essential for producing lignin‐based value‐added materials. Herein, we modify the PL properties of lignin via genetic engineering of novel luminophore structures. Feruloyl‐CoA 6′‐hydroxylase (F6′H1) is a 2‐oxoglutarate‐dependent dioxygenase that catalyses the conversion of feruloyl‐CoA, an intermediate of the biosynthesis pathway of monolignol, into 6′‐hydroxyferuloyl‐CoA, the precursor of scopoletin. To modify the lignin PL properties, the F6′H1 gene (*F6′H1*) from *Arabidopsis thaliana* is overexpressed in the hybrid aspen ( *Populus tremula* × *tremuloides* T89), incorporating scopoletin into the lignin molecule. Cellulolytic enzyme lignin (CEL) was isolated from transgenic aspens with different overexpression levels of *F6*′*H1* and evaluated for its PL properties. In *N.N*‐dimethylformamide solution, CEL from the *F6*′*H1*‐overexpressed aspen emitted clear PL with higher intensity and a longer wavelength than the wild‐type CEL. Size exclusion chromatography revealed a wide molar mass distribution of the chromophore. Interestingly, the PL of the CEL from the F6′H1 transgenic lines was limitedly quenched in low polar solvents and at high concentrations. The CEL from F6'H1 emitted obvious PL not only in solution but also in polymer film. Furthermore, the CEL of F6′H1 lines exhibited a reversible photodimerisation reaction characteristic of coumarins. These results suggest that genetic engineering can incorporate new luminophores such as scopoletin into lignin, thus producing value‐added materials.

## 1 Introduction

**FIGURE 1:**
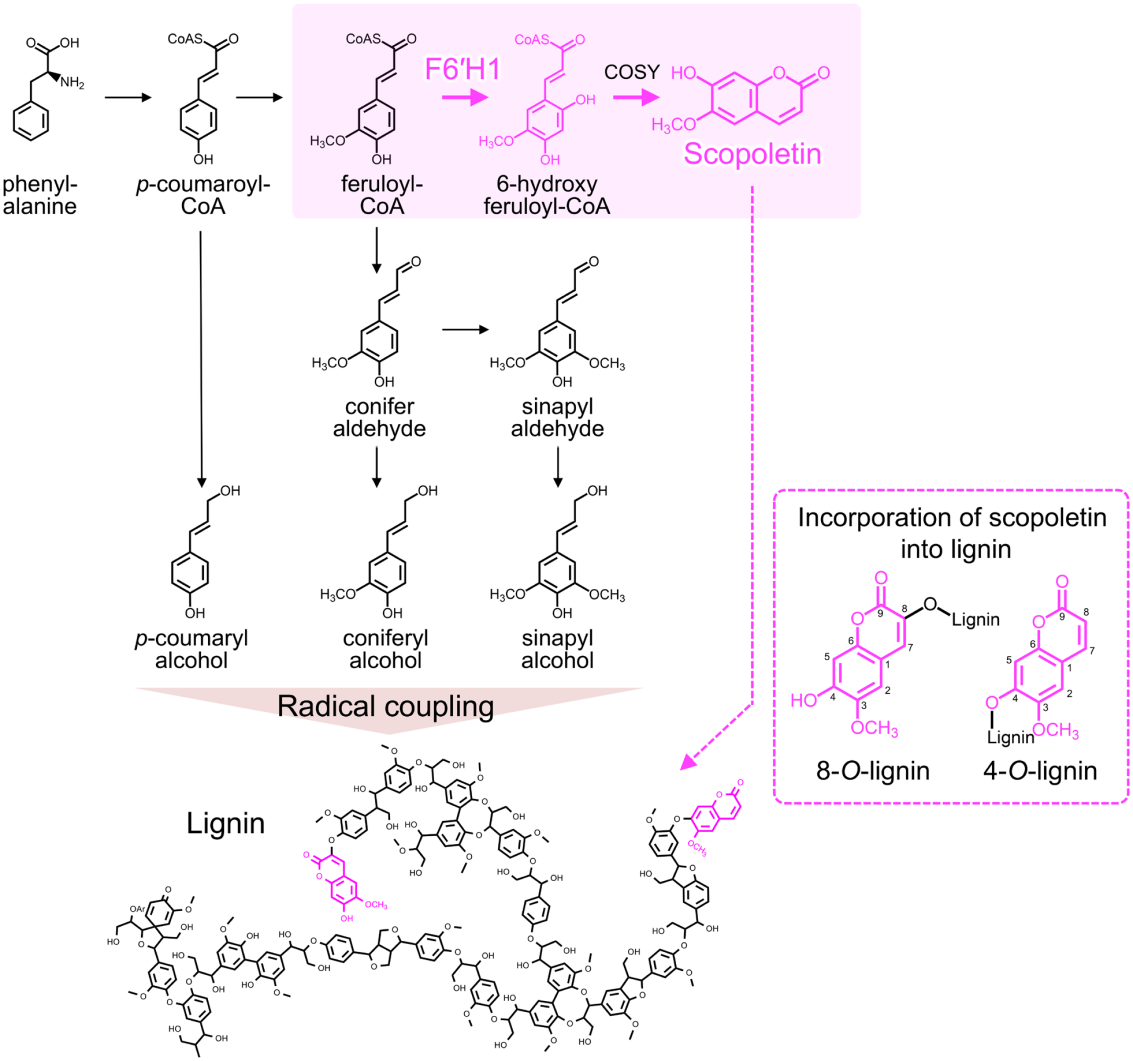
Monolignol biosynthesis pathway and the conversion of feruroyl‐CoA into scopoletin catalysed by F6′H1.

Carbon‐neutral woody biomass has been targeted as an alternative resource to fossil fuel resources in sustainable societies. Lignin, a cell wall component of woody biomass, is the most abundant natural aromatic polymer on Earth and is expected as a feedstock for value‐added materials. Lignin is synthesised via the oxidative coupling reactions of three monolignols: *p*‐coumaryl, coniferyl and sinapyl alcohols, which are produced via the phenylpropanoid pathway (Figure 1). The intricate chemical structure of lignin contributes to its poor degradability and processability, thereby reducing the efficiency of downstream processes such as enzymatic saccharification and chemical pulping. To enhance lignin degradability, various strategies have been explored to modify its chemical structure, including shortening lignin chains, reducing cross‐linking with carbohydrates, increasing hydrophilicity and incorporating labile linkages (de Vries et al. 2021). In particular, genetic engineering technology can qualitatively and quantitatively modify the properties of lignin, achieving efficient utilisation of polysaccharides by the overexpression or reduction of genes involved in the biosynthesis pathway of monolignol. For instance, the ferulate‐5‐hydroxylase gene, which catalyses the hydroxylation of coniferyl alcohol and coniferaldehyde in the biosynthesis of syringyl (S) lignin units, has been overexpressed to increase the syringyl‐to‐guaiacyl ratio in transgenic plants, achieving a significantly higher pulping yield and glucose release than wild‐type plants (WT) after kraft pulping and enzymatic saccharification following liquid hot water treatment, respectively (Huntley et al. 2003; Li et al. 2010). Furthermore, the resulting S‐rich lignin can be efficiently depolymerised into monomeric products via formaldehyde‐stabilised hydrogenolysis (Shuai et al. 2016). The cinnamyl alcohol dehydrogenase gene, encoding the enzyme that catalyses the final step of lignin monomer biosynthesis, often leads to the change in lignin structure, resulting in an increase in the efficiency of enzymatic hydrolysis (Van Acker et al. 2017) and kraft pulping (Lapierre et al. 1999). Another approach involves the truncation of side chains in lignin monomers, which reduces the degree of polymerisation and facilitates enzymatic saccharification (Eudes et al. 2012). Target‐specific miRNAs have successfully down‐regulated various laccases in poplars, reducing lignin polymerisation and improving the efficiency of cell wall glycation (Guo et al. 2023). Alternatively, the biosynthesis of catechyl (C) lignin, which has a structurally homogeneous and linear polymer, has been targeted through genetic engineering, owing to its superior processability compared to conventional lignin. Notably, successful induction of C lignin biosynthesis was achieved in transgenic tracheary elements of *Pinus radiata* via suppression of *caffeoyl‐CoA O‐methyltransferase* (*CCoAOMT*) expression (Wagner et al. 2011) and in *Medicago truncatula* through combined suppression of *caffeic acid O‐methyl transferase (COMT)* and *CCoAOMT*, resulting in up to 15% C lignin accumulation (Ha et al. 2023). Multiple studies have demonstrated that C lignin can be efficiently depolymerised through hydrogenolysis to yield a single C type monomer with high selectivity, owing to its non‐condensed benzodioxane structure (Li et al. 2018, 2023; Stone et al. 2018; Wang et al. 2021).

There have been numerous studies reporting the use of genetic engineering techniques to modify lignin structure with the aim of improving its processability and degradability. In contrast, there are few examples of applying genetic approaches to enhance its functionality as a macromolecule—for instance, for its antioxidant properties, amphiphilicity or optical characteristics such as ultraviolet (UV) absorption and photoluminescent (PL).

In a previous study, various genes involved in the enzyme‐catalysed biosynthesis of lignin were overexpressed in *Arabidopsis* under the control of the xylem‐preferential cinnamate 4‐hydroxylase (C4H) promoter. These genes were subsequently screened for their potential to induce structural modifications in lignin (Sakamoto et al. 2020). Overexpression of the feruloyl‐CoA 6′‐hydroxylase 1 gene (*F6′H1*) did not significantly alter the lignin content or affect the growth of transgenic *Arabidopsis* but successfully accumulated scopoletin in the cell wall. F6′H1 is a 2‐oxoglutarate‐dependent dioxygenase that catalyses the conversion of feruloyl‐CoA, an intermediate in the lignin biosynthesis pathway, to 6‐hydroxyferuloyl CoA. Feruloyl‐CoA undergoes spontaneous isomerisation and lactonisation reactions or is further processed to scopoletin by coumarin synthase (COSY) (Figure 1). Simultaneous overexpression of the *F6′H1* and *COSY* genes also lead to scopoletin accumulation in the lignin of *Arabidopsis* (Hoengenaert et al. 2022). Furthermore, Wang et al. overexpressed *F6′H1* in the hybrid aspen ( *Populus tremula* × *tremuloides* T89), successfully accumulating and incorporating scopoletin into the lignin molecule (Wang et al. 2025). As scopoletin is clearly fluorescent, it would impart a unique PL property to scopoletin‐incorporated lignin. The present study investigates the influence of scopoletin incorporation on the PL properties of lignin. Cellulolytic enzyme lignins (CELs) were extracted from the WT and two F6′H1 transgenic lines and their PL properties were evaluated in various solvents [*N*,*N*‐dimethylformamide (DMF), ethanol, chloroform (CHCl_3_), *n*‐hexane and water] and two polymers [poly (methyl methacrylate) (PMMA) and polyvinyl alcohol (PVA)].

Furthermore, coumarin exhibits a unique photo‐reactivity property called photo‐dimerisation, which can be reversed under irradiation at a specific wavelength. More specifically, coumarin can be dimerised at wavelengths longer than 300 nm and reverted to a monomer at wavelengths shorter than 300 nm (Cazin et al. 2021). This unique photo‐property has been exploited in coumarin‐containing polymers for electro‐optical studies, photo‐reversible systems, coumarin‐containing biopolymers, polymerisations, chiral stationary phases in high‐performance liquid chromatography (HPLC) and fluorescent tags and fluoroprobes (Trenor et al. 2004). For example, PMMA has been copolymerised with coumarin to create thermo‐ and photo‐responsive polymers (Okada and Sato 2021), and methacrylate bearing a 2‐ethylhexyl plasticising group has been copolymerised with a photoactive coumarin unit to fabricate a three‐dimensional shape‐memory polymer (Tarek Benkhaled et al. 2022). Coumarin polymer‐grafted nanocellulose can form high‐performance, photo‐responsive barrier layers (Vijay et al. 2022). Herein, we hypothesise a unique photoreactivity, similar to that of coumarin‐containing polymers, in our transgenic aspen lignin. We also study the photo‐reactivities of CELs from the WT and F6′H1 lines.

Since lignin possesses a large molar extinction that can potentially be exploited in lignin‐based optical materials, lignin has been added as a UV absorber in polymers (Sadeghifar and Ragauskas 2020; Zhang and Naebe 2021), and incorporated into a lignin‐based fluorescence sensor. Furthermore, the fluorescent property of lignin enables fluorescence microscopy observations of the lignin distribution within wood cell walls (Donaldson 2013; Donaldson et al. 2010). Recently, detailed lignin distributions have been revealed by advanced fluorescence‐based microscopy technologies such as fluorescence lifetime imaging microscopy and Förster resonance energy transfer (Chabbert et al. 2018; Escamez et al. 2021; Terryn et al. 2018). Previously, we tuned the intensity and wavelength of lignin photoluminescence by changing the lignocellulose species, extraction method and environmental media (solvents and polymers) (Takada et al. 2022). The PL property can be utilised in fluorescence reagents, fluorescence sensors and spectral conversion agents. Herein, we introduce new luminophores into lignin molecules using a genetic engineering approach. In contrast to the conventional genetic engineering strategies aimed at modifying lignin structure, the application of such techniques specifically designed to exploit lignin as a polymer material has seldom been applied, and the present study is the first reported strategy worldwide that aims to modify PL properties through the introduction of luminophores. In the present study, the incorporation of structural units with unique functionalities not only enhanced the polysaccharide recovery rate as previously reported (Wang et al. 2025) but also revealed a novel potential for the utilisation of polymeric lignin as an optical material.

## 2 Results and Discussion

### 2.1 Lignin Extracted From F6′H1 Overproduced Aspen

Scopoletin accumulation in the transgenic lines was evaluated using pyrolysis gas chromatography–mass spectrometry (Py‐GC/MS) (Figure S1). The lines with “high” accumulation levels (#10 and #16) showed severe dwarfism and were omitted owing to their limited sample number and infeasibility for practical implementation. Therefore, the WT (“no” detection level), line #12 (“low” accumulation level) and line #6 (“medium” accumulation level) were selected for the present analysis.

**FIGURE 2:**
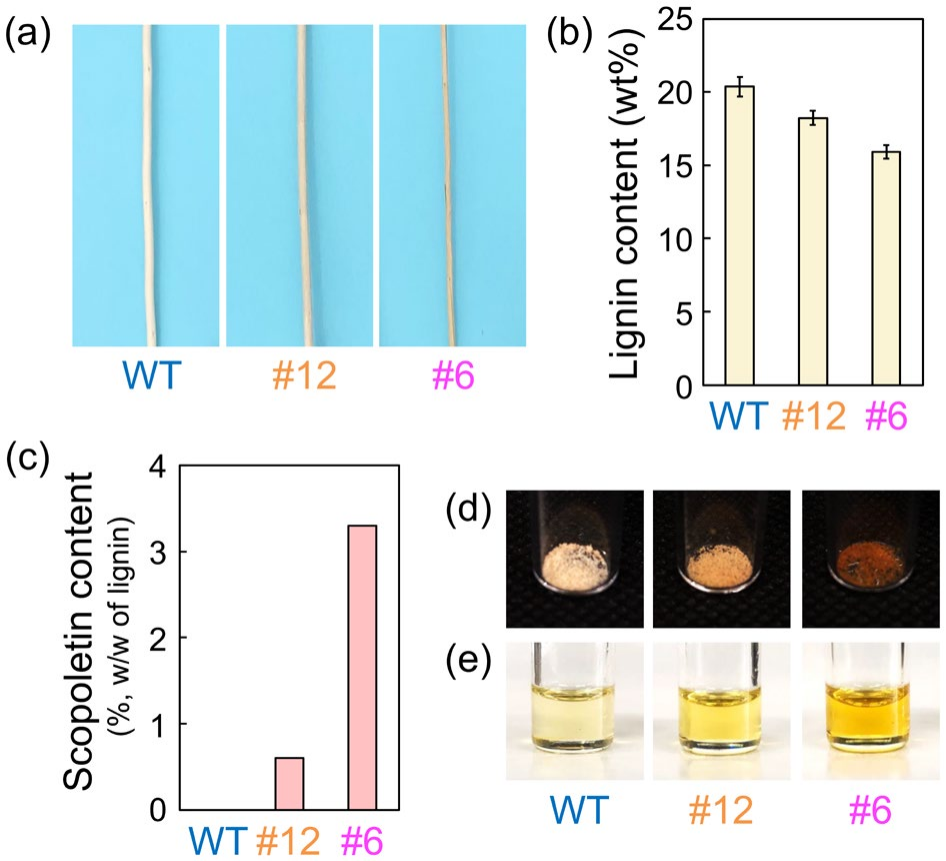
(a) Photographs of the debarked wood xylem from the WT, F6′H1#12 and F6′H1#6 plant lines and (b) lignin contents of their extractive‐free wood powders; (c) amounts of incorporated scopoletin (detected by Py‐GC/MS); (d) photographs of cellulolytic enzyme lignins (CELs) extracted from the WT, F6′H1#12 and F6′H1#6 lines and (e) their DMSO solutions (10 mg mL^−1^).

Photographs of the debarked wood xylem from the WT, F6′H1#12 and F6′H1#6 lines are shown in Figure 2a. The wood xylem of the highly overexpressed line (F6′H1#6) appears red, the common colour of lignin‐modified transgenic plants (Lapierre et al. 2004; Nabuqi et al. 2020). The lignin content was slightly lower in the F6′H1 lines than in the WT line (Figure 2b). The Py‐GC/MS analysis detected scopoletin‐derived peaks in the F6′H1 lines (Figure 2c). As the scopoletin monomer metabolite was carefully removed by solvent extraction prior to all analyses, the detected scopoletin was obviously incorporated into the lignin molecule. Scopoletin incorporation in lignin molecules has been previously confirmed by heteronuclear single quantum correlation nuclear magnetic resonance (Wang et al. 2025) and likely occurs via ether linkages, as shown in Figure 1.

To analyse the PL properties in the transgenic aspens, CELs were extracted from each line. The F6′H1 CEL powder and their dimethyl sulfoxide (DMSO) solutions were differently coloured like the debarked wood xylem (Figure 2d,e). The dark colour of the highly overexpressed lines indicates an alteration of the chromophores in the F6′H1 lignin.

### 2.2 UV–Vis Absorbance and PL Properties of the CELs in Solution

**FIGURE 3:**
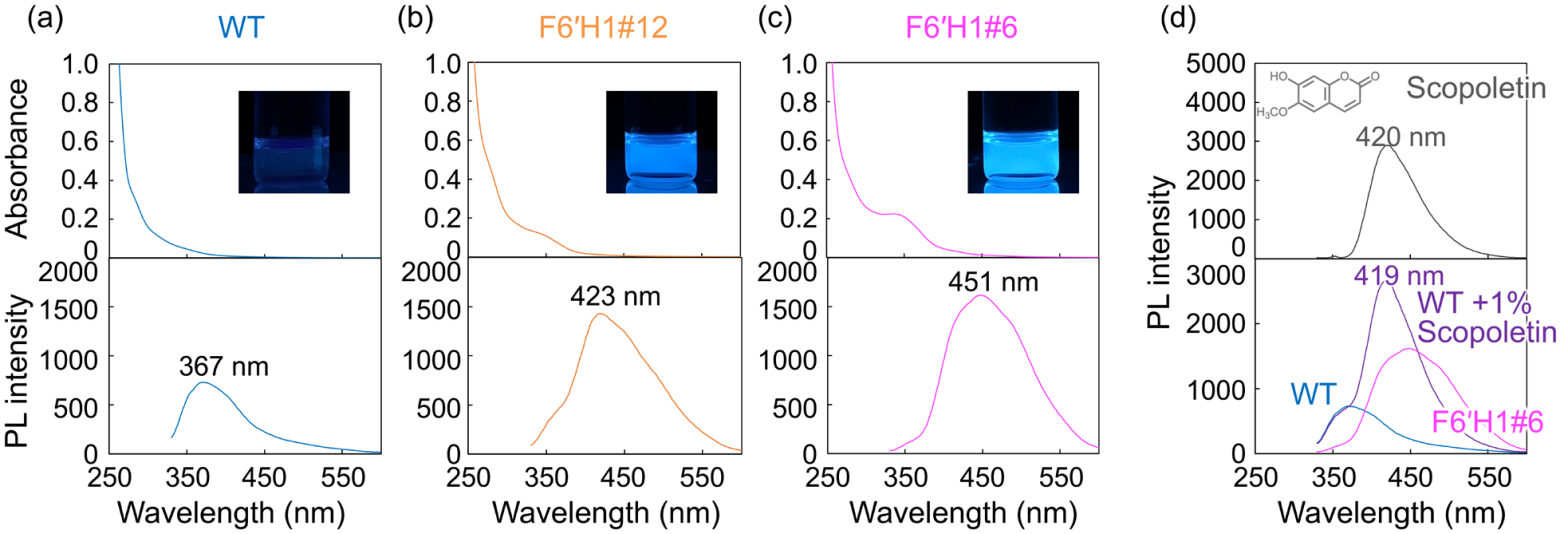
UV–Vis absorption (top) and PL spectra (bottom) of the (a) WT, (b) F6′H1#12 and (c) F6′H1#6 CELs dissolved in DMF (0.1 mg mL^**−**1^) and excited at 320 nm. The photographs (insets) were taken under 365 nm irradiation. (d) PL spectra of scopoletin (top) in DMF solution (0.001 mg mL^**−**1^) and the mixed WT CEL/scopoletin in DMF solution (bottom).

Panels (a)–(c) of Figure 3 compare the UV–Vis absorption and PL spectra of the CELs in 0.1 mg mL^−1^ DMF solution. The WT CEL solution exhibits an absorption shoulder at 280 nm and a clear PL peak at 367 nm under 320 nm UV light (Figure 3a). The spectra of the F6′H1 lines clearly differed from those of the WT. The absorption peaks at 350 nm were consistent with the UV spectrum of scopoletin (Figure S2) and were thus attributed to the scopoletin structure. The PL spectra of both F6′H1#6 and #12 peaked with higher intensities at longer wavelengths than the WT. The photographs (insets) demonstrate notable fluorescence from the CELs in solution. The spectra of F6′H1#6 particularly deviated from the WT spectra, with a peak at 451 nm under the same excitation wavelength. After adding 10% of the scopoletin monomer, the PL spectrum of the WT CEL in DMF solution resembled that of F6′H1#12 under 320 nm irradiation (Figure 3d) but quite differed from that of F6′H1#6. Such a wavelength shift can be induced by formation of an excimer, an unstable excited molecule created by the combination of two monomers. Excimer formation can also be detected as a difference in the PL lifetime *τ*. As the PL lifetimes did not significantly differ between the WT (*τ* = 2.52 ns) and F6′H1#6 (*τ* = 2.50 ns) (see Table S1), excimer formation cannot be the cause of the wavelength shift in F6′H1#6. Instead, the luminophore is newly formed by the incorporation of scopoletin into lignin.

The F6′H1 lines emitted high‐intensity PL at other excitation wavelengths (280, 350 and 400 nm; see Figure S3). Under 280 nm excitation, the DMF solution of the WT CELs exhibited clear PL with a peak at 345 nm. In the PL spectrum of the F6′H1#12 CEL, the peak at 345 nm decreased and a new PL peak appeared at 421 nm. In the PL spectrum of the F6′H1#6 CEL, the peak at 345 nm disappeared, and a single broad peak appeared at 447 nm. The decreased PL at 345 nm is due to self‐absorption by scopoletin, which strongly absorbs 345 nm UV light. The PL spectra of the WT and F6′H1 lines obviously differed under 350 and 400 nm excitation, with F6′H1#6 showing a higher PL intensity than F6′H1#12. Apparently, increasing the amount of incorporated scopoletin improved the PL properties.

**FIGURE 4:**
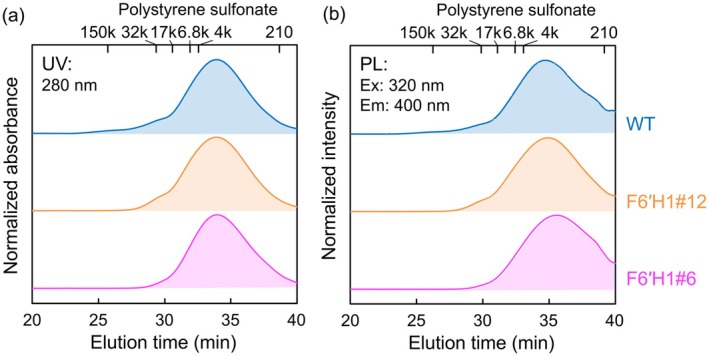
SEC chromatograms of CELs from the WT, F6′H1#12 and F6′H1#6 lines in 10 mM LiBr DMSO solution: (a) UV absorbance detected at 280 nm, and (b) PL intensity excited at 320 nm and detected at 400 nm.

The molar mass distributions of the luminophores in the CELs were evaluated using SEC equipped with UV and PL detectors. The molar mass distribution of lignin is often calculated using UV detection data, assuming a homogeneous coefficient factor. In the calculation calibrated by a series of polystyrene sulfonate standards, the F6′H1#6 (Mw = 3460) and #12 (Mw = 5680) yielded slightly lower molar mass distributions than the WT (Mw = 8790) (Figure 4a and Table S2). This result is reasonable because scopoletin is an end unit of the lignin molecule (Wang et al. 2025). In contrast, the PL spectrum obtained under 320 nm excitation and 400 nm detection reveals that the luminophores in all CELs are composed of a wide range of molar masses rather than low‐molar mass compounds. As the solution is too lowly concentrated for aggregation, this condition eliminates PL quenching.

**FIGURE 5:**
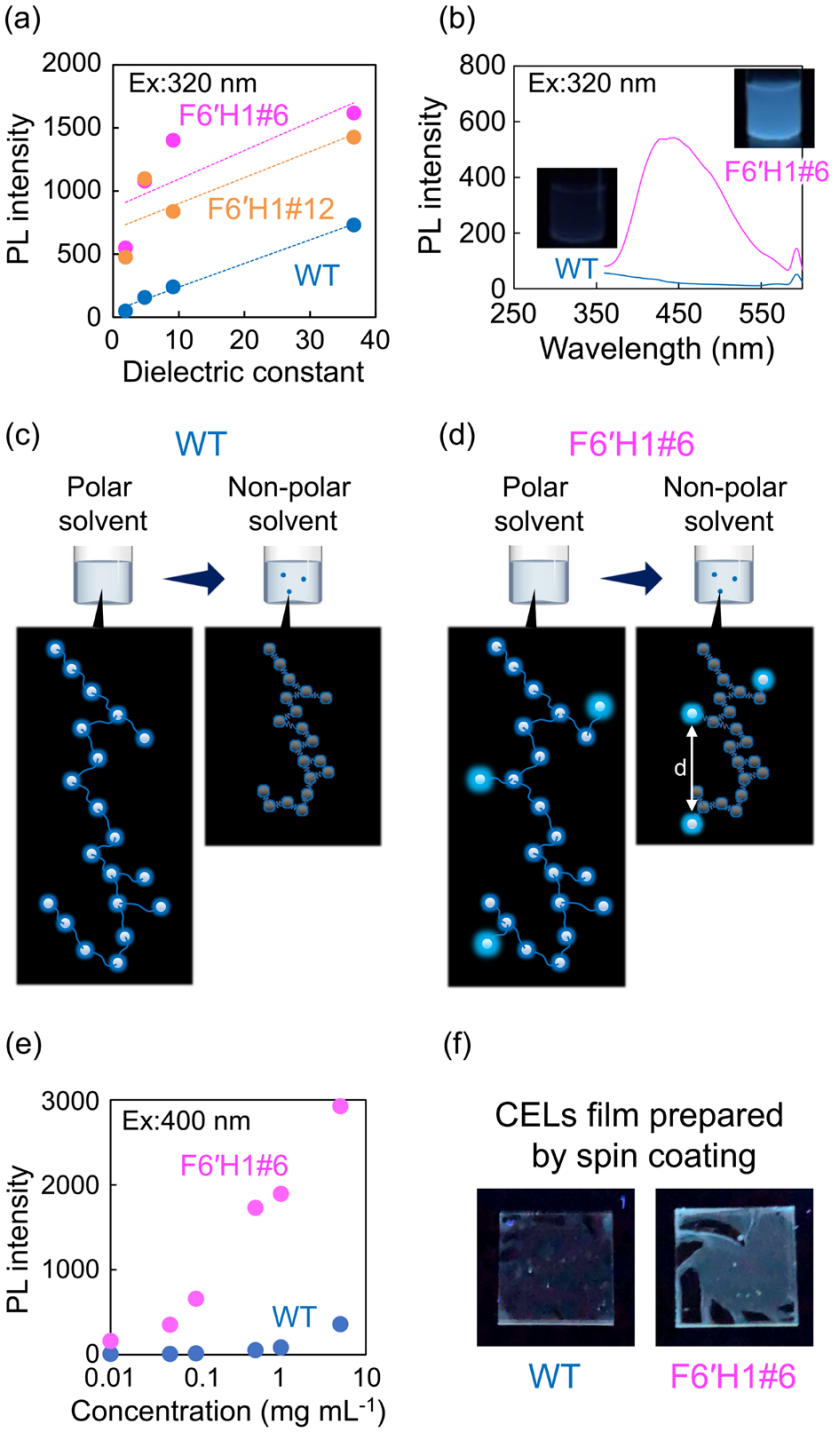
(a) Correlation between the maximum PL intensity of the CEL solutions (0.1 mg mL^**−**1^, excited at 320 nm) and solvent dielectric constant; (b) PL spectra of the CELs from WT and F6′H1#6 in *n*‐hexane, and photographs of the solution after dispersion by sonication (insets); images of the proposed luminophore distributions in (c) WT and (d) F6′H1#6 CELs in polar and non‐polar solvents; (e) correlation between the maximum PL intensity (excited at 400 nm) and CEL concentration in DMF solution; (f) photographs of CEL films prepared via solvent removal using spin coating (excited at 365 nm).

To study the effect of solvents on the PL properties of the CELs, the UV–Vis and PL spectra of the CELs were analysed in dichloromethane (*ε* = 9.1), CHCl_3_ (*ε* = 4.8), and *n*‐hexane (*ε* = 1.9) (Figure S4). The dielectric constants (summarised in Table S3) indicate the hydrophobicities of the solvents. The WT CEL was quenched in the hydrophobic solvents (dichloromethane and CHCl_3_), as evidenced by the positive correlation between the solvent dielectric constant and PL intensity (Figure 5a). The lignin in the WT CEL aggregates in hydrophobic solvents, decreasing the distance between the luminophores and leading to quenching (Takada et al. 2022). In contrast, the CELs of the F6′H1 lines showed limited quenching in hydrophobic solvents. Surprisingly, the CEL of F6′H1#6 showed a clear PL even in low‐polarity *n*‐hexane (*ε* = 1.9), in which it is lowly soluble and tends to aggregate (Figure 5b).

From these findings, we can discuss the distribution of the luminophore (i.e., the scopoletin moiety) within the lignin molecule. As scopoletin is incorporated into lignin at the end‐point via ether linkages and it is distributed over a wide range of molecular weights, it is unlikely to be gathered and distributed at specific points in the lignin molecule. Figure 5c shows the proposed scopoletin distribution based on the scopoletin content in lignin (0.6%–3.6%). As the scopoletin moieties are separated, the CELs of the F6′H1 lines undergo limited quenching in hydrophobic solvents where aggregates are easily formed. Quenching is an obstacle in practical applications of PL lignin, as explained in the Introduction. The present F6'H1 lines can overcome this obstacle by maintaining suitable distances between their homogeneously distributed luminophores.

To check the unquenching phenomena of the F6′H1 lines in DMF solution, we studied the influence of CEL concentration on the PL properties (Figures 5e and S5). Under high‐concentration conditions, the PL spectra excited at 320 or 350 nm were strongly affected by self‐absorption; thus, only the PL spectra excited at 400 nm are shown. The F6′H1#6 CEL emitted clear PL even in 5 mg mL^**−**1^ DMF. In addition, the solvents were removed using a spin coater under extremely high‐concentration conditions, forming ultrathin lignin films. The films emitted clear PL under UV light irradiation (Figure 5f). Although the CEL powder produced no luminescence, the ultrathin films prepared via spin coating luminesced even in the absence of a surrounding medium. These results indicate a limited quenching phenomena in F6′H1, as illustrated in Figure 5c.

### 2.3 UV–Vis Absorbance and PL Properties of the CELs in Aqueous Solution

**FIGURE 6:**
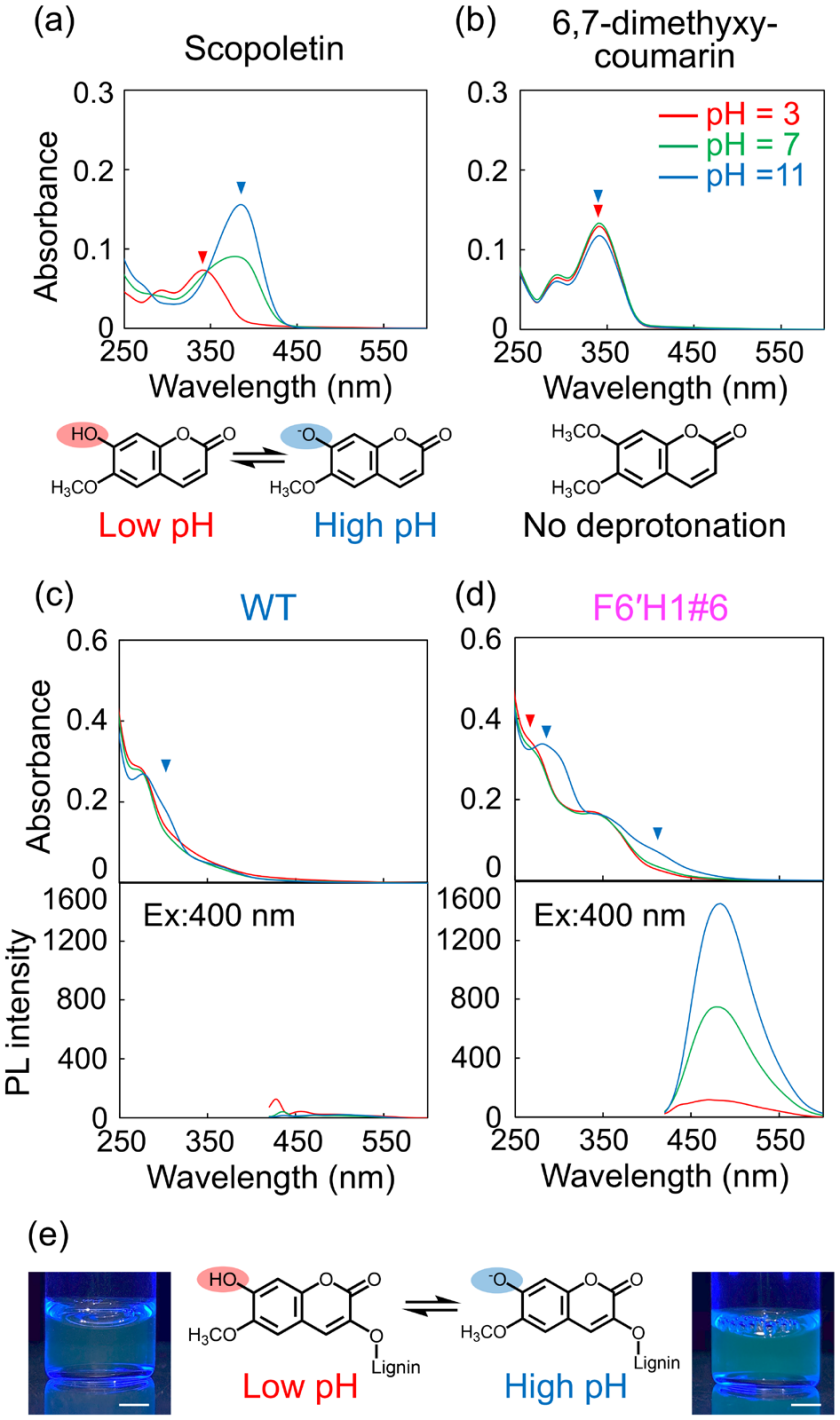
Influence of solution pH on the UV–Vis absorption and PL spectra in solution: UV–Vis absorption spectra of (a) scopolotin and (b) 6,7‐dimethxylcoumarin solution at different pH levels (pH = 3, 7, 11); UV–Vis and PL spectra of (c) WT CEL and (d) F6′H1#6 CEL solutions at different pH levels (pH = 3, 7, 11). The PL spectra were exited at 400 nm. (e) Photographs of the F6′H1#6 CEL in aqueous solution irradiated at 365 nm, and illustrations showing the deprotonation of the scopoletin moiety of lignin. The scale bars in photographs indicate 5 mm.

This subsection discusses the PL spectra in aqueous solution. As scopoletin has a phenolic hydroxyl group, the aqueous‐solution pH is expected to affect the UV–Vis absorbance and PL spectra of the CELs. The UV absorption peak appeared at 340 nm under the acidic condition (pH = 3) but shifted to 385 nm under the alkaline condition (pH = 11) (Figure 6a, arrows) due to deprotonation of the hydroxyl group. This peak shift under different pH conditions was not observed in methylated scopoletin (6,7‐dimethoxy‐coumarin), which contains no hydroxyl groups (Figure 6b), but was observed in the CEL of the F6′H1 lines, probably because part of the scopoletin incorporated into the F6′H1 lignin possesses a phenolic hydroxyl group (Figure 1). Panels (c) and (d) of Figure 6 show the spectra of the CELs from WT and F6′H1#6, respectively, in aqueous solution. In the UV–Vis spectrum of the WT CEL, the absorbance at 300 nm slightly increased under alkaline conditions, which is again explained by deprotonation of the hydroxyl group of the lignin molecule. In contrast, the UV–Vis spectrum of the F6′H1#6 CEL displays a new peak at 300 nm and a notable increase in the 400 nm peak. Apparently, pH exerts a higher influence on the UV–Vis absorption of F6′H1#6 than on the UV–Vis absorption of WT. The PL spectrum of the F6′H1#6 CEL solution was even more sensitive to pH, showing a nearly 10‐times higher PL intensity under the alkaline condition than under the acidic condition. These notable changes in the UV–Vis spectra and PL intensity suggest the presence of a phenolic hydrogen group on part of the scopoletin incorporated into F6′H1 lignin (see Figure 6e). A similar trend was observed in an organic solvent while changing the pKa of the solution by adding a base (acetoamide; see Figure S6). In the F6′H1#6 CEL solution alone, the UV–Vis absorbance at 450 nm slightly increased with increasing loading amount of acetoamide.

### 2.4 UV–Vis and PL Properties of the CELs in Polymer

**FIGURE 7:**
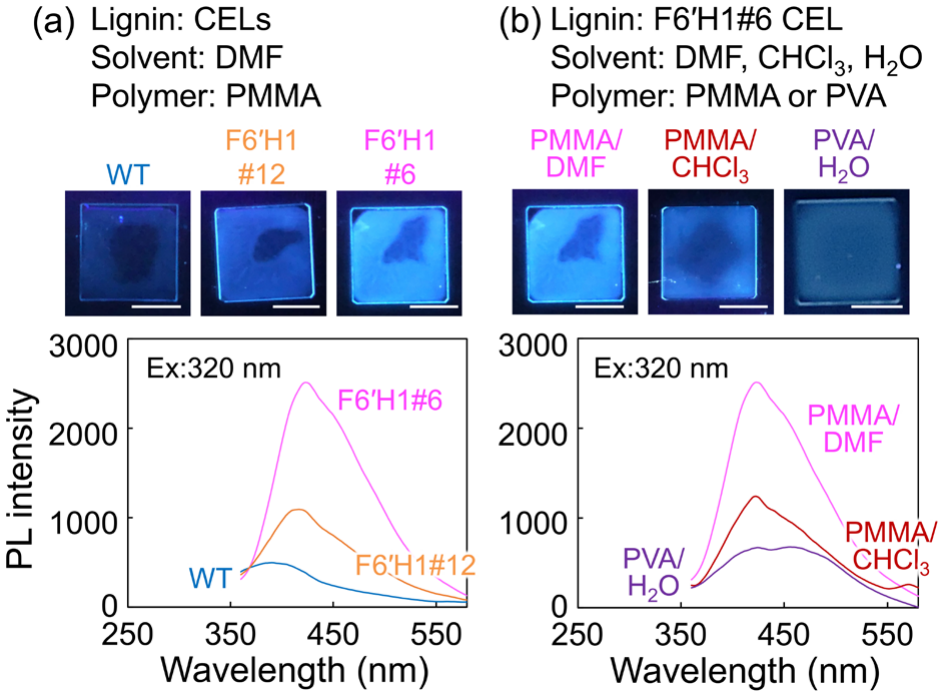
PL spectra and photographs of the films containing CEL: (a) PL spectra of WT and F6′H1 CEL films prepared with PMMA and DMF, and (b) PL spectra of three F6′H1#6 CEL films prepared with various solvents and polymers. The PL spectra were excited at 320 nm and the photographs were taken at 365 nm. The scale bars in photographs indicate 1 cm.

The CELs were mixed with a PMMA solution and then cast onto quartz slides to obtain transparent films. The PL spectra of the films under 320 nm excitation are shown in Figure 7a. All CEL‐containing films emitted clear PL, although the PL intensity was highest in the F6′H1#6 film, as similarly observed in solution. This difference in PL intensity is evident in photographs of the films (Figure 7a). Owing to its relatively low polarity, PMMA dissolves in both DMF and CHCl_3_. Therefore, to deduce the effect of solvent on the PL properties of the films, PMMA films were prepared in DMF and CHCl_3_ solutions (Figure 7b). The two PMMA films produced notably different spectra; in particular, the DMF preparation emitted higher‐intensity PL than the CHCl_3_ preparation, reflecting the PL‐intensity trend in solution. A transparent film prepared from hydrophilic PVA also showed a clear PL property, but the shapes and intensities of its PL spectrum notably differed from those of the PMMA films (Figure 7b), indicating that the properties of the polymer also affect the PL spectra.

### 2.5 Photodimerisation of Coumarin‐Incorporated Lignin

**FIGURE 8:**
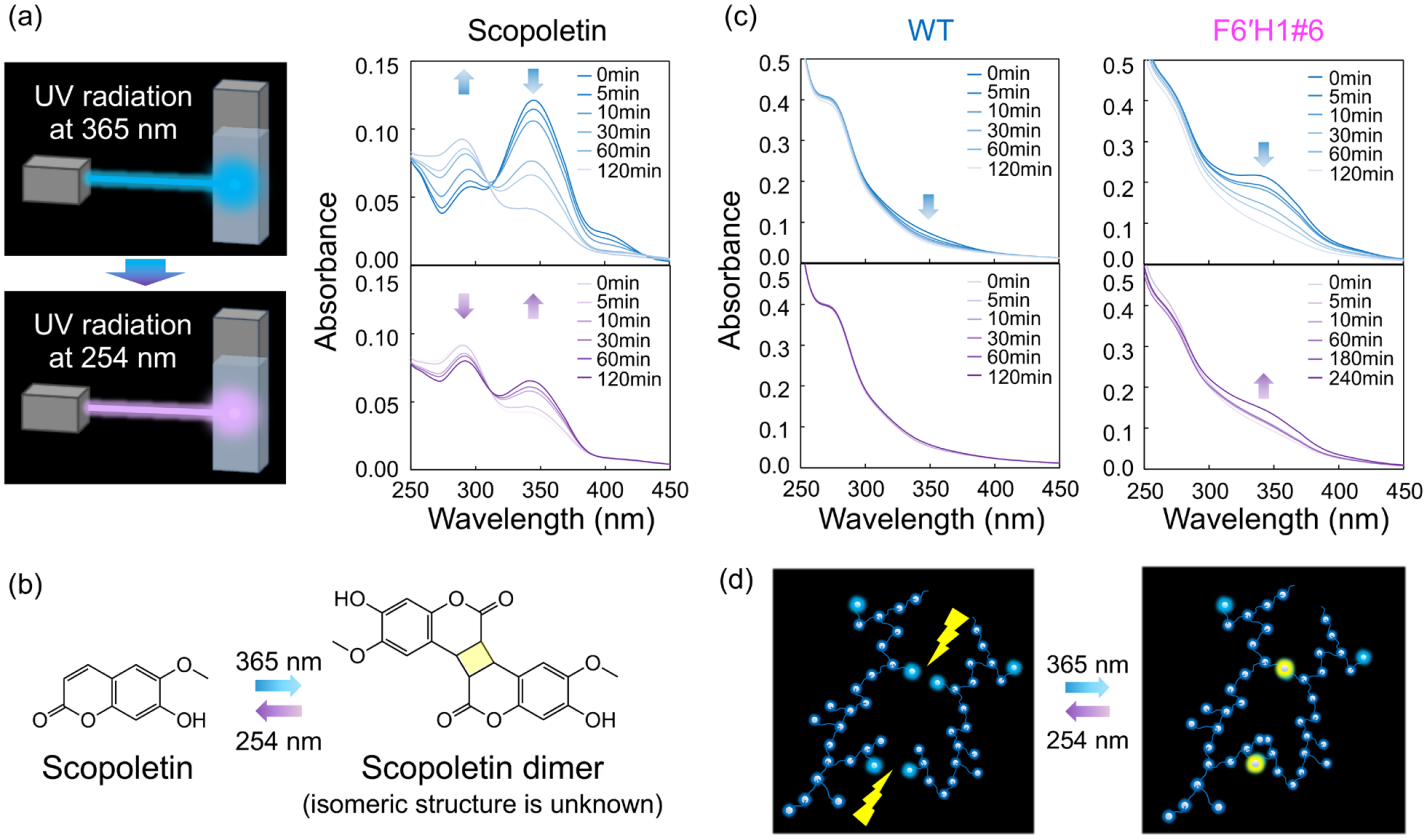
(a) Photodimerisation reactions of scopoletin. Changes in the UV–Vis absorption spectra of scopoletin in ethanol solution under 365 nm UV irradiation for 0–120 min (top) followed by 254 nm radiation for 0–120 min (bottom); (b) the photodimerisation reaction of scopoletin; (c) changes in the UV–Vis absorption spectra of WT and F6′H1#6 CELs in ethanol solution under 365 nm UV irradiation for 0–120 min (top) followed by 254 nm irradiation for 0–120 or 0–240 min (bottom); (d) photodimerisation reaction of the scopoletin moiety in the F6′H1#6 CEL.

Finally, the photodimerisation of scopoletin was evaluated using UV–Vis spectroscopy. Under irradiation at 365 nm for 120 min, the peaks at 350 and 330 nm in the UV–Vis spectrum of the scopoletin–ethanol solution gradually decreased and increased, respectively (Figure 8a). Next, the photo‐reacted solution was irradiated at 254 nm for 120 min. The UV–Vis spectrum gradually returned to that of the original scopoletin, with an increase and decrease in the peaks at 350 and 330 nm, respectively. Such a reversible change in UV–Vis absorbance has been reported in coumarins (Obi et al. 1999), suggesting that scopoletin was photodimerised in the present system (Figure 8b). Coumarin photodimerisation yields four possible products: *syn* head‐to‐head, *syn* head‐to‐tail, *anti* head‐to‐head and *anti* head‐to‐tail. The selectivity depends on factors such as the distances between double bonds and solvent polarity (Cazin et al. 2021). The isomeric structure of the scopoletin dimer is not elucidated here.

Figure 8c shows the results of the same experiment on the WT and F6′H1#6 CELs in ethanol solution. The UV–Vis spectrum of the WT CEL in ethanol showed a slight reduction of the 350 nm absorption peak under 365 nm irradiation for 120 min, but was unchanged after irradiating the photo‐reacted solution at 254 nm for 120 min. In contrast, the UV–Vis spectrum of the F6′H1#6 CEL in ethanol was obviously reduced after 120 min of 365 nm irradiation. Furthermore, when the photo‐reacted solution was irradiated at 254 nm for 120 min, the UV–Vis spectra gradually returned to the original spectrum with an increased peak intensity at 350 nm. This reversible change in the UV–Vis absorbance suggests photodimerisation of the scopoletin moiety in F6′H1#6 lignin. To our knowledge, photodimerisation from lignin (a natural aromatic polymer material) has not been previously reported. This photodimerisation‐mediated reversible reaction is potentially exploitable in electro‐optical studies, photo‐reversible systems, coumarin incorporation into biopolymers, polymerisation, chiral stationary phases for HPLC, fluorescent tags, fluoroprobes, tissue engineering, drug delivery systems, soft robotics and four‐dimensional printing (Cazin et al. 2021). Although the radiation wavelength and light intensity must be further optimised in applications, scopoletin‐incorporated lignin is a potentially valuable feedstock of functional value‐added materials. Furthermore, photodimerisation forms one of four isomeric products depending on the structure of the coumarins and the surrounding medium (e.g., the solvent). Therefore, a genetic‐engineering strategy that introduces various coumarins to control the reactivity and selectivity of photodimerisation is expected.

The analysis of PL properties clarified the photo‐reactivity and other interesting PL properties of scopoletin‐incorporated lignin. The saccharification efficiencies of the F6′H1 lines exceeded that of the WT line even without pretreatment (Wang et al. 2025), indicating that polysaccharide components can be easily processed in the F6′H1. As discussed above, lignin is potentially convertible into functional materials, implying that cell wall components can be comprehensively utilised.

## 3 Experimental Procedures

### 3.1 Biomass and Chemicals

*Agrobacterium*‐mediated transformation was performed in the WT hybrid aspen ( *Populus tremula* × *tremuloides* T89) as previously described (Wang et al. 2025). The transgenic plants were propagated in Murashige Skoog medium. Rooted plantlets were cultured in the same medium for 4 weeks and then transferred to pots with soil, where they were cultivated over 10 weeks at 25°C under a 16/8 h light/dark cycle of fluorescent light in a climate‐conditioned room. After 13 weeks of growth, the stems of the resulting eight lines were debarked and then dried in the shade (Figure S7). For each transgenic line, 3 to 5 individual plants were generated and subjected to structural analysis. For PL spectral analysis, representative individuals showing average characteristics were selected, and CEL was isolated. PL measurements and photodimerisation reactions were performed three times to confirm reproducibility. The most representative spectra are presented in the figures.

All chemicals were of reagent grade and used without further purification. PMMA (average MW: 120 000) and PVA (completely hydrolyzed, average DP: 500) were purchased from Sigma‐Aldrich Co. LLC.

### 3.2 Lignin Content, Accumulation of Scopoletin and Isolation of CELs

The debarked stems from the three lines (WT, F6**′**H1#12 and F6′H1#6) were ground in a milling machine (MultiBeads Shocker MB1200C, Yasui Kikai, Osaka, Japan) and sieved to obtain fine particles sized less than 150 μm. To prepare the extractive‐free wood powder, the milled powder was soaked in a 50 mM NaCl aqueous solution overnight and washed with water. After extraction with methanol, acetone, ethanol (twice), ethanol/toluene (1:2, v/v, twice) and acetone, the wood powder was air‐dried at room temperature, and its lignin content was quantified using the acetyl bromide method (Iiyama and Wallis 1988). The scopoletin accumulated in the cell wall was confirmed using Py‐GC/MS as previously reported (Wang et al. 2025). The CELs were prepared as described in the literature (Madigal et al. 2025).

### 3.3 UV–Vis and PL Spectra of Isolated Lignin in Solution

The isolated CEL was dissolved in 10 mg mL^−1^ DMSO. The solution was then diluted in various solvents: DMF, ethanol, dichloromethane, CHCl_3_ and *n*‐hexane (0.1 mg mL^−1^). The UV–Vis absorption and PL spectra were recorded with V‐730 (Jasco Co. Ltd., Tokyo, Japan) and FP‐8200 (Jasco Co. Ltd., Tokyo, Japan) instruments, respectively. For analysis in aqueous solutions, the prepared lignins were dissolved in acidic (pH = 3) and alkaline (pH = 11) solutions of hydrochloric acid and sodium hydroxide, respectively, prepared at 0.1 mg mL^−1^. The fluorescence lifetimes of the lignin solutions were analysed using a light pulser (C10196, Hamamatsu Photonics K.K.; Hamamatsu, Japan) and a streak scope (C4334, Hamamatsu Photonics K.K.) with excitation and detection wavelengths of 373 and 440 nm, respectively.

### 3.4 Molar Mass Distribution of the Luminophores

The molar mass distribution was analysed using size exclusion chromatography (SEC) with two columns (PolarGel M columns, 7.5 × 300 mm) and a guard column (7.5 × 50 mm) at 40°C. The eluent solvent was 10 mM LiBr DMSO solution (0.5 mL min^−1^). The lignin (0.24 mg mL^−1^) was prepared in 10 mM LiBr DMSO. Two detectors were employed: a UV detector with a wavelength of *λ* = 280 nm and a fluorescence detector with excitation and detection wavelengths of 320 and 400 nm, respectively. The SEC system (Chromaster, Hitachi Ltd., Tokyo) was calibrated with standard polystyrene sulfonate (Sigma‐Aldrich Japan, Tokyo) standards of molar masses ranging from 210 to 150 000.

### 3.5 Preparation of Lignin‐Containing Transparent Films

The CELs were dissolved in 1,4‐dioxane with 5% DMSO. The resulting solution (10 μL) was mounted on the cover glass, and the solvent was removed by spin coating at 2000 rpm for 10 s, forming a lignin film. This step was performed three times to ensure a sufficient quantity of lignin.

To prepare lignin‐containing transparent films, PMMA was dissolved in 100 mg mL^−1^ of DMF or CHCl_3_ and PVA (100 mg mL^−1^) was dissolved in water. Then, 1 mL of the desired polymer solution was mixed with 10 μL of various CELs dissolved in 10 mg mL^−1^ DMSO. The mixtures were cast onto quartz slides and dried under air to obtain transparent films. The UV–Vis and PL spectra of the transparent films were analysed similarly to the solutions.

### 3.6 Photodimerisation Reaction in the Solution

The photodimerisation reaction was performed in ethanol solvent. The scopoletin ethanol solution (0.01 mg mL^−1^) or CEL ethanol solution (0.1 mg mL^−1^) was irradiated with 365‐nm light in the FP‐8200 instrument (bandwidth 20 nm). After the designated reaction time, the UV–Vis absorbance spectra were analysed using the V‐730 instrument (Jasco Co. Ltd.). The reverse reaction was similarly performed under UV irradiation (254 nm).

## Author Contributions

M.T. and S.K. conceived the study. M.T. and S.H. performed most of the experiments and data analysis. N.W. and M.U. prepared and maintained the transgenic plants. M.T. managed the study and manuscript submission. M.T., S.H. and S.K. drafted the manuscript. All authors reviewed and approved the final version of the manuscript.

## Conflicts of Interest

The authors declare no conflicts of interest.

## Supporting information


**Figure S1:** The relative area ratio of scopoletin to coniferyl alcohol, detected using Pyrolysis‐gas chromatography/mass spectrometry (Py‐GC/MS) in the CWR of F6′H1 transgenic lines.
**Figure S2:** UV–Vis spectra of scopoletin monomer in DMF, ethanol and CH_2_Cl_2_ solution at 0.01 mg mL^−1^.
**Figure S3:** PL spectra of DMF solution of CELs from WT and F6′H1 transgenic lines at 0.1 mg mL^−1^ excited at 280 nm, 350 nm and 400 nm.
**Figure S4:** (a–c) PL spectra of WT and F6′H1#6 CELs in various solvents (DMF, Ethanol, CH_2_Cl_2_, CHCl_3_) excited at 320 and 350 nm.
**Figure S5:** PL spectra of WT and F6′H1#6 CELs in DMF solution at a variety of concentration (0.01 to 5 mg mL^−1^) excited at 400 nm.
**Figure S6:** UV–Vis spectra of (a, c) WT‐3 and (b, d) F6′H1#6 CELs in DMSO at the concentration of 0.1 mg mL^−1^ with addition of different amount (0, 0.1, 1, 10 μL) of acetoamide.
**Figure S7:** Plants were grown in pots for 15 weeks in a conditioned culture room.
**Table S1:** Fluorescence life time of CELs in DMSO at the concentration of 0.1 mg mL^−1^.
**Table S2:** Mw calculated from SEC chromatographs of CELs in 10 mM LiBr DMSO solution.
**Table S3:** Dielectric constant of various solvents used in this study.

## Data Availability

The data that supports the findings of this study are available in the Supporting Information of this article.
