# Supplementary material for: Photoluminescence Properties of Lignin With a Genetically Introduced Luminophore in a Transgenic Hybrid Aspen That Overproduces Feruloyl‐CoA 6′‐Hydroxylase
Source: Plant Biotechnol J. 2025 Oct 3;24(2):905–14. doi: 10.1111/pbi.70390 (PMC12906806; doi:10.1111/pbi.70390)
Supplement: Supplementary file 1 — Figure S1: The relative area ratio of scopoletin to coniferyl alcohol, detected using Pyrolysis‐gas chromatography/mass spectrometry (Py‐GC/MS) in the CWR of F6′H1 transgenic lines. Figure S2: UV–Vis spectra of scopoletin monomer in DMF, ethanol and CH2Cl2 solution at 0.01 mg mL−1. Figure S3: PL spectra of DMF solution of CELs from WT and F6′H1 transgenic lines at 0.1 mg mL−1 excited at 280 nm, 350 nm and 400 nm. Figure S4: (a–c) PL spectra of WT and F6′H1#6 CELs in various solvents (DMF, Ethanol, CH2Cl2, CHCl3) excited at 320 and 350 nm. Figure S5: PL spectra of WT and F6′H1#6 CELs in DMF solution at a variety of concentration (0.01 to 5 mg mL−1) excited at 400 nm. Figure S6: UV–Vis spectra of (a, c) WT‐3 and (b, d) F6′H1#6 CELs in DMSO at the concentration of 0.1 mg mL−1 with addition of different amount (0, 0.1, 1, 10 μL) of acetoamide. Figure S7: Plants were grown in pots for 15 weeks in a conditioned culture room. Table S1: Fluorescence life time of CELs in DMSO at the concentration of 0.1 mg mL−1. Table S2: Mw calculated from SEC chromatographs of CELs in 10 mM LiBr DMSO solution. Table S3: Dielectric constant of various solvents used in this study. [file PBI-24-905-s001.docx]

**Supporting information for:**

Photoluminescence properties of lignin with a genetically introduced luminophore in a transgenic hybrid aspen that overproduces feruloyl-CoA 6′-hydroxylase

**Authors:**

Masatsugu Takada,^a,b^* Shota Horinouchi,^a^ Naning Wang,^a^ Mikiko Uesugi,^a^ and Shinya Kajita^a^

a. Graduate School of Bio-Applications and Systems Engineering (BASE), Tokyo University of Agriculture and Technology, 2-24-16, Nakacho Koganei, Tokyo 184-8588, Japan.

b. Graduate School of Agriculture, Ehime University, 7-5-3, Tarumi Matsuyama, Ehime 790-8566, Japan. Email: takada.masatsugu.qb@ehime-u.ac.jp

Figure S1 The relative area ratio of scopoletin to coniferyl alcohol, detected using Pyrolysis-gas chromatography/mass spectrometry (Py-GC/MS) in the CWR of F6′H1 transgenic lines. The transgenic lines were categorized into low, medium, and high ranges in accordance with the relative area index. The star marked lines (WT, F6′H1#12, and F6′H1#6) were used in this study. Adapted from Wang et al., Frontiers in Plant Science, 2025, 16, 1543168, under a Creative Commons CC BY license.

Figure S2 UV-Vis spectra of scopoletin monomer in DMF, ethanol and CH_2_Cl_2_ solution at 0.01 mg mL^-1^

Figure S3 PL spectra of DMF solution of CELs from WT and F6′H1 transgenic lines at 0.1 mg mL^-1^ excited at 280 nm, 350 nm and 400 nm.

Figure S4 (a-c) PL spectra of WT and F6′H1#6 CELs in various solvents (DMF, Ethanol, CH_2_Cl_2_, CHCl_3_) excited at 320 and 350 nm.

Figure S5 PL spectra of WT and F6′H1#6 CELs in DMF solution at a variety of concentration (0.01 to 5 mg mL^-1^) excited at 400 nm.

Figure S6 UV-Vis spectra of (a,c) WT-3 and (b,d) F6′H1#6 CELs in DMSO at the concentration of 0.1 mg mL^-1^ with addition of different amount (0, 0.1, 1, 10 μL) of acetoamide.

Figure S7 Plants were grown in pots for 15 weeks in a conditioned culture room. Scale bar indicates 20 cm. The star marked lines (WT, F6′H1#12, and F6′H1#6) were used in this study. Adapted from Wang et al., Frontiers in Plant Science, 2025, 16, 1543168, under a Creative Commons CC BY license.

Table S1 Fluorescence life time of CELs in DMSO at the concentration of 0.1 mg mL^-1^

| Sample | Fluorescence life time (ns) |
| --- | --- |
| WT | 2.52 |
| F6′H1#12 | 2.46 |
| F6′H1#6 | 2.50 |
| Scopoletin | 4.52 |

Table S2 Mw calculated from SEC chromatographs of CELs in 10 mM LiBr DMSO solution. Detectors are UV at 280 nm and PL excited at 320 nm and detected at 400 nm.

| Sample | Mw | |
| --- | --- | --- |
|  | UV | PL |
| WT | 8,790 | 6,210 |
| F6′H1#12 | 5,680 | 3,880 |
| F6′H1#6 | 3,460 | 2,110 |

Table S3 Dielectric constant of various solvents used in this study

| Solvent | Dielectric constant |
| --- | --- |
| Water | 80.4 |
| DMF | 36.7 |
| Ethanol | 24.0 |
| Dichloromethane | 9.1 |
| Chloroform | 4.8 |
| *n*-Hexane | 1.9 |
